# SIA Vector Analysis and Keratometry Prediction in Temporal SICS with 350 µm Scleral Incision Depth

**DOI:** 10.22336/rjo.2026.34

**Published:** 2026

**Authors:** Prabhakar Srinivasapuram Krishnacharya, Kedar Janardhan Prabhu, Shreya Nataraj, Anisha Cyprian, Preethi Prasanna Belagutti

**Affiliations:** Department of Ophthalmology, JSS Hospital, Mysore, Karnataka, India

**Keywords:** temporal SICS, scleral incision depth, horizontal keratometry, vertical keratometry, SIA vector, SICS = small incision cataract surgery, ATR = against the rule, WTR = with the rule, Kh = horizontal keratometry, Kv = vertical keratometry, D = diopter

## Abstract

**Objectives:**

The magnitude and axis of surgically induced astigmatism depend on the sclera-corneal tunnel dimensions. Often, the incision depth is subjective, and standardization is rarely emphasized. This study attempts to evaluate a fixed scleral incision depth of 350 µm at 1-week post-op. The aim is to determine the SIA vector and centroid using the Pythagorean theorem and keratometric measurements.

**Materials and methods:**

This study was conducted from January to July 2025 and recruited 42 patients at a tertiary health institution. All patients received scleral incisions to a depth of 350 µm after preoperative auto-keratometry measurements. Preoperative astigmatism was classified into WTR, ATR, and oblique types.

**Results:**

The overall mean age was 67.14 (± 9.23) years, with 27 (64.29%) males and 15 (35.71%) females. A positive correlation (r = 0.93, 0.69) was found for pre- and post-operative keratometry. T-test for pre- and post-operative vertical keratometry revealed statistical significance (p < 0.0009). The mean SIA magnitude and axis were 2.05 (± 1.75) D and 77.94º (± 59.68º), respectively. The Wilcoxon signed-rank test showed statistical significance for SIA magnitude (p=0.0003). The centroid was at 22.3º. A 28.57% conversion rate from ATR to WTR was observed, with 71.43% remaining unchanged postoperatively. High prediction accuracy was observed for preoperative horizontal and vertical keratometry (R^2^ values of 0.93 and 0.74, respectively).

**Discussion:**

This work adds a unique dimension to the literature by explicitly linking a fixed tunnel depth to the SIA vector and keratometric prediction. These results provide a platform for future research exploring scleral incision depth as a determinant of astigmatic outcomes.

**Conclusions:**

This study finds a statistically significant difference in vertical keratometry with moderate SIA magnitude. Astigmatic axis transition was unchanged in most patients, likely due to a standardized scleral incision depth. Centroid demonstrated a minimal SIA magnitude on horizontal keratometry. An excellent postoperative keratometry prediction was determined.

## Introduction

Temporal small-incision cataract surgery (SICS) is advantageous for enhanced surgical field exposure and for inducing minimal postoperative astigmatism. An increased incidence of surgically induced astigmatism was reported in 48.28% with the superior incision approach compared with the temporal approach [1]. A higher rate of with-the-rule astigmatism (WTR) was found following temporal SICS [2]. A precisely preset scleral incision depth to a specific measurement may influence postoperative refractive outcomes.

Incision depth of less than 399 microns, measured by AS-OCT, showed fewer astigmatic changes than a depth of more than 400 microns [3].

This study calculated the SIA vector using Pythagoras’ theorem in the Cartesian coordinate system of analysis, rather than the subtraction method for pre- and postoperative keratometry values described by previous authors [4]. Pawar et al. studied surgically induced astigmatism using a readily accessible MS Excel-based SIA calculator (version 2.0), which is freely available [5].

The accuracy of postoperative keratometric prediction was assessed at 1 week post-op using a linear regression algorithm with preoperative keratometry values, and the model was evaluated using performance metrics. The novelty of this study lies in investigating the SIA vector with a standardized scleral incision depth and postoperative keratometry prediction.

## Methods and materials

This prospective study was conducted from January to July 2025 and enrolled 42 patients at a tertiary health institution. Patients were enrolled according to inclusion and exclusion criteria after institutional ethical clearance. All types of cataracts were included, except in patients with prior ocular surgery or inflammatory ocular disorders that may affect scleral incision remodeling.

Pre-operative workup consisted of documenting the demographic profile, pre-operative diagnosis, and laterality of the disease, along with Snellen’s visual acuity and rebound tonometry recordings. Biometry included autorefractokeratometry, axial length, anterior chamber depth, and lens thickness measurements. Autorefractokeratometry was performed at post-op 1 week, in contrast to a previous study that measured using a manual Bausch and Lomb keratometer [6].

A straight scleral incision was made, similar to the previous study, and the nucleus was delivered by visco-expression in all patients after hydro-dissection and hydro-delineation [7,8]. The coding for SIA vector calculations was performed in Python using a Google Colab notebook. Necessary libraries were imported, such as pandas for exploratory data preprocessing and NumPy for mathematical and trigonometric functions.

### SIA vector calculation methods

The magnitude of pre- and postoperative astigmatism was calculated by subtracting the horizontal corneal curvatures from the vertical corneal curvatures. Pre- and post-operative axes were converted from degrees to radians because the Python interactive environment can recognize and analyze them in radians. And multiplied by 2 because the astigmatism is symmetrical.

Cartesian coordinates for x and y were calculated for pre- and post-operative curvatures in vector form. x (pre) = pre-operative cylinder * Cos (ø),

y (pre) = pre-operative cylinder * Sin (ø),

x (post) = post-operative cylinder * Cos (ø),

y (post) = post-operative cylinder * Sin (ø).

X and Y components of SIA vectors were calculated by subtracting pre-operative vectors from the corresponding post-operative vectors.

The magnitude (length) of the SIA vector was calculated as the square root of the sum of the squares of the two orthogonal astigmatic X and Y components. Finally, the axis (direction) of the SIA vector was calculated using the arctan2 function, and the angle was halved modulo 180º to obtain the original axis in the standard 0 to 180-degree notation.

### Keratometry prediction analysis methods

The prediction model was constructed by applying a linear regression algorithm to training data with pre-operative corneal curvatures as features, with and without outliers. Preoperative corneal curvatures were defined as the X (independent) variable and postoperative corneal curvatures as the Y (target or dependent) variable. After allocating 80% of the data for training and 20% for testing, with a fixed random state for reproducibility, the linear regression function was imported from the sklearn linear model. Both univariate and multivariate linear regression analyses were performed.

The linear regression model was then fitted to train the X and Y data. The trained regression model was used to make predictions on the X testing set. The performance of the model was evaluated by mean absolute error (MAE), mean squared error (MSE), root mean squared error (RMSE), and R-squared (Coefficient of Determination). Finally, the best linear regression model, which achieved a high R2, was built using the model intercept and coefficients.

## Results

The present prospective study recruited 42 patients with an overall mean age of 67.14 (± 9.23) years, ranging from 39 to 82 years. Continuous variables were analyzed as mean and standard deviation. Categorical variables were computed as proportions. Descriptive statistics were performed for overall age, gender-wise age distribution, and keratometry measurements. The chi-square test was performed to assess the statistical significance of the categorical variables. Paired t-test was done on continuous variables for statistical significance set at a significance level of p-value less than 0.05.

Pearson correlation coefficients were calculated for pre- and post-operative keratometry measurements. Histograms were plotted to assess the normality of the distribution. Box-and-whisker plots were constructed to identify outliers in the dataset. Scatter plots were designed to visualize pre- and post-operative keratometry measurements and assess linear distribution.

Based on the Pythagorean principle, SIA vectors were calculated to determine SIA magnitude and axis. Histograms, box-and-whisker plots, and scatter plots were constructed to examine the magnitude and axis of the SIA. Polar plots were built for quick visualization and interpretation of the SIA vector. The mean SIA vector was calculated and depicted as a centroid vector in the single- and double-angle polar plots.

Univariate and multivariate regression analyses were performed to predict post-operative keratometry. Consequently, predictive regression equations were calculated. Metrics were used to evaluate the performance of the models. The Shapiro-Wilk test was used to assess normality, and the Wilcoxon signed-rank test was used to analyze pre- and post-operative keratometry.

## Discussion

The present prospective study recruited 42 patients with an overall mean age of 67.14 (± 9.23) years, suggesting a common age for the development of cataract, in contrast to Kumar N et al.’s study, which reported a mean age of 59.8 years but with a similar male predominance. And the induced astigmatism was calculated by subtracting the flatter dioptric keratometry value from the dioptric value of the steeper meridian. No statistically significant difference was observed in the gender-wise age distribution in the present study (Table 1).

**Table 1:** Descriptive statistics for overall age and gender wise age distribution

n =42	Overall (years)	Males (years)	Females (years)
Mean	67.14	65.81	69.53
Standard deviation	± 9.23	± 9.02	± 9.44
Minimum	39	39	57
Maximum	82	79	82
Count (Percent)	42 (100%)	27 (64.29%)	15 (35.71%)

The median postoperative horizontal keratometry did not change after temporal SICS; however, the median postoperative vertical keratometry increased by a diopter. The median postoperative keratometric axis did not change but extended from 60 to 145 degrees compared to the preoperative astigmatic axis that ranged from 60 to 110 degrees (Table 2).

**Table 2:** Descriptive statistics for pre- and postoperative keratometry

n = 42	Preop kh (Diopter)	Preop kv (Diopter)	Preop axis (Degrees)	Postop kh (Diopter)	Postop kv (Diopter)	Postop axis (Degrees)
Mean	43.98	44.86	86.07	43.79	45.83	98.45
Standard deviation	± 1.59	± 1.54	± 38.78	± 1.86	± 2.42	± 55.58
Minimum	40.25	40.75	5	37.75	38.5	10
Maximum	47	47.5	170	47	50.75	180

The histogram for pre-operative horizontal keratometry followed a normal Gaussian distribution, and the postoperative histogram showed a right skew deviation due to one outlier at the flattest keratometry measurement. The histogram of pre- and post-operative vertical keratometry showed a right-skewed distribution due to an outlier; however, the post-operative keratometry measurements followed a normal distribution. The histogram of the post-operative astigmatic axis revealed that 43.75% of patients measured 175 degrees, probably due to standardized scleral incision depth.

**Fig. 1:**
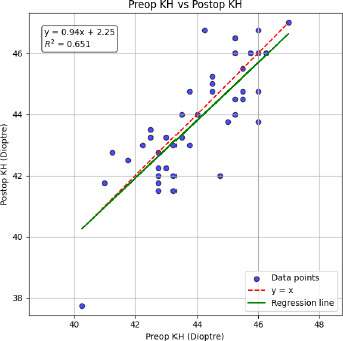
Positive linear association of pre- and postoperative horizontal corneal curvatures

**Fig. 2:**
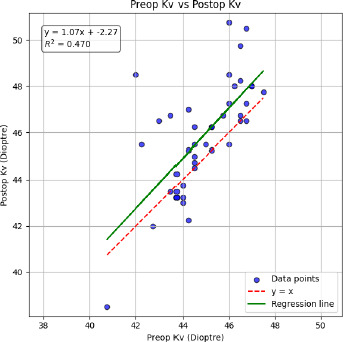
Positive linear association of pre- and postoperative vertical corneal curvatures

The scatter plot revealed a positive linear association for both pre- and post-operative keratometry. A coefficient of determination (R^2^) of 0.65 implies 65.10% of variance in horizontal keratometry, which is explained by pre-operative horizontal keratometry (Fig. 1). Similarly, the coefficient of determination (R^2^) for vertical keratometry was 0.46 (Fig. 2). Both preoperative corneal curvatures were significant predictors of postoperative keratometric values (p < 0.00). The scatter plot illustrating pre- and post-operative axis distributions is inappropriate, since the astigmatic axis follows a circular distribution.

An excellent positive correlation (r = 0.94) was observed between the two preoperative corneal curvatures, indicating a linear effect. A good positive correlation (r = 0.81) was observed for pre- and post-operative horizontal keratometry, but not for vertical keratometry (r = 0.69). A moderate negative correlation (r = -0.29) was observed between pre- and post-operative astigmatic axes, implying a decrement effect.

Pre- and post-operative vertical keratometry showed a statistically significant difference of a diopter with anticlockwise axis rotation, possibly due to the torque effect induced by the temporal scleral incision. No statistically significant difference was noted between the pre- and postoperative axes, suggesting a plausible role for depth standardization. Furthermore, most preoperative astigmatic types (73.80%) remained unchanged, while 26.20% exhibited an axial transition in the first postoperative week, providing evidence for incision depth optimization (Table 3). Overall, after temporal SICS, astigmatism was equally redistributed between with- and against-the-rule astigmatism, in contrast to previous studies that reported a shift towards with-the-rule astigmatism [8,9].

**Table 3:** The postoperative redistribution of preoperative astigmatism

n = 42	Pre-operative astigmatism (per cent)	Post-operative redistribution (per cent)
With the rule of astigmatism	28 (66.8)	19 (45.2)
Against the rule of astigmatism	08 (19.0)	16 (38.1)
Oblique astigmatism	06 (14.2)	07 (16.7)
Axis transition	11 (26.20%)	
Axis non-transition	31 (73.80%)	

Reddy et al.’s study on 64 patients reported a rule astigmatism of 1.57 (± 0.24) diopters at 6 weeks by the subtraction method, in contrast to 2.24 (± 1.86) diopters found in the present study one week postop [10]. Zawar SV et al. reported rule astigmatism in 93.8% and against-the-rule astigmatism in 6.2% of eyes at 6 weeks following temporal SICS, compared with 45.23% and 38.09%, respectively, in the present study. Kavitha K et al. studied 100 patients and reported vertical meridian flattening and horizontal meridian steepening with an ATR shift following a superior incision. Vice versa for a temporal approach, aligning with the present study, which found a WTR astigmatism transition [11]. Rajappa et al.’s study reported an average SIA magnitude of 0.43 (± 0.13) diopters at 1 week postop, compared with 1.28 (± 0.68) diopters in the present study. Interestingly, the author reported 45.9% of against-the-rule astigmatism using the SIA calculator version 2.1, similar to the present study’s results.

The mean and standard deviation of SIA magnitude were 1.28 (± 0.68) diopters after eliminating outliers, which aligns with previous publications; however, the authors used the SIA calculator version 2.1, a Microsoft Excel program [12,13]. Chatterjee T et al. investigated 100 patients and reported a mean SIA of 1.09 (± 0.42) diopters in the temporal group, which surprisingly correlated well with the present study’s mean SIA of 1.28 (± 0.68) diopters, without outliers [7] (Table 4).

**Table 4:** Descriptive statistics of the SIA vector magnitude and axis

N = 42	SIA magnitude with outliers (Diopter)	SIA magnitude without outliers (Diopter)	SIA axis with outliers (Degrees)	SIA axis without outliers (Degrees)
Mean	2.09	1.28	77.93	78.27
Standard deviation	± 1.75	± 0.68	± 59.68	± 60.91
Minimum	0.00	0.00	0.00	0.00
Maximum	7.46	2.51	178.91	178.91

**Fig. 3:**
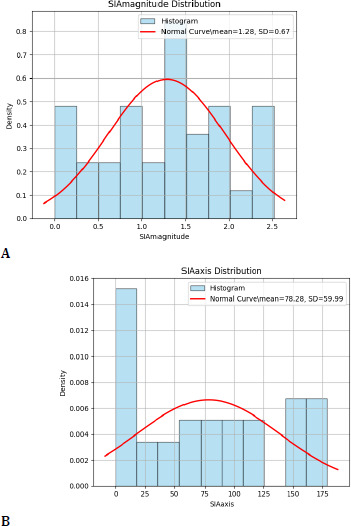
A, B Histograms for SIA magnitude and axis after outlier elimination

The mean and standard deviation for the SIA axis were similar: 70.00 (± 60.00) degrees, unaffected by outliers. The histogram of SIA magnitude followed a normal distribution, with 40% (0.5 x 0.8) of patients achieving an astigmatism magnitude of 1.00 to 1.50 diopters. The SIA axis showed 37.5% (25 x 0.015) of patients redistributing at the 180-degree astigmatic axis, primarily due to the preset depth of 350 microns (Fig. 3 A, B).

**Fig. 4:**
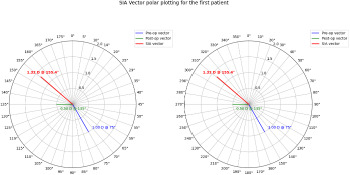
Single and double angle method for SIA vector polar plotting for the first study patient

The SIA vector was calculated by subtracting the pre-operative SIA vector from the postoperative SIA vector. An appropriate clinical method for plotting the SIA vector is polar plotting, as it depicts the SIA magnitude and angle simultaneously and clarifies the absolute change in the SIA vector. The length of the SIA vector denotes the astigmatism magnitude expressed in diopters, while the direction of the SIA vector indicates the astigmatic axis expressed in degrees. The magnitude of the SIA vector is estimated across concentric circles in 0.5-diopter intervals and axes at 5- and 10-degree spacing, as shown in the first and second diagrams (Fig. 4, Table 5).

**Table 5:** The SIA vector analysis results at post-op one week

n = 42	Values
Mean pre-operative astigmatism (D)	0.88 (± 0.55)
Mean post-operative astigmatism (D)	2.05 (± 1.75)
95% CI (SIA magnitude) (D)	1.55 to 2.64
SIA Centroid vector	0.52 D at 22°
95% CI (centroid magnitude) (D)	0.17 to 1.20
95% CI (centroid axis)	117.5° to 107.0°
p-value (Shapiro–Wilk test)	0.0001
p-value (Wilcoxon signed-rank test)	0.0003

A single-angle vector plot is essentially a clinical representation of the change in induced astigmatism across 180 degrees. In comparison, a double-angle vector plot spans 360 degrees, which accurately represents symmetrical astigmatism. The first diagram shows a single-angle vector representation of the current study patient, with the SIA vector at 155.4 degrees; the same angle doubles to 310.8 degrees in a double-angle vector representation, since astigmatism is always symmetrical. Goel et al.’s prospective study included 40 eyes and analyzed SIA Vector at 12 weeks using the double-angle method. Interestingly, the overall mean age and gender-wise age distribution in the present study were in line with those of this study. And reported mean astigmatism reduction from 1.70 (± 0.50) to 0.92 (± 0.45) diopters after temporal incision [14].

**Fig. 5:**
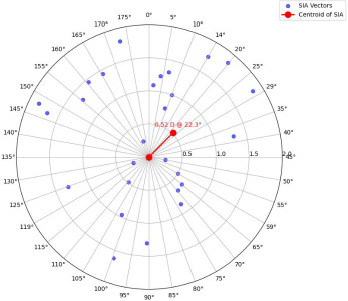
Clinical representation of the centroid SIA vector at the first week postoperative

The centroid was the mean of all individual SIA vectors shown in the polar plot. Blue dots represented the SIA vectors for all patients, and the red line indicated the centroid’s magnitude and axis. This study reported an induced magnitude of 0.52 diopters and an induced axis of 22.3 degrees, suggesting minimal change in surgically induced astigmatism. And 22.3 degrees of axis suggested a possible shift or rotation of the induced astigmatic axis towards the 180-degree meridian, corresponding to the horizontal corneal curvature, by the temporal approach (Fig. 5).

Arthur E et al. studied 58 patients with preoperative ATR astigmatism. They investigated SIA using vector analysis of Cartesian coordinates at 12 weeks, similar to the present study but with a superior approach. And reported a centroid of 1.42 diopters at 179º, 2.48 diopters at 0.00º, and 1.07 diopters at 1.00º, for pre- and postoperative vector and SIA vector, respectively, aligning with the present study of 0.59 diopters at 70.62º, 2.25 diopters at 93.75º, and 0.52 diopters at 22.3 degrees [15].

The keratometric prediction models further underscore the clinical relevance of scleral incision-depth architecture. Horizontal keratometry demonstrated robust predictive power, with an accuracy of 0.75, even in the presence of outliers. This indicated that the primary effect of the temporal incision was directed along the horizontal meridian, producing consistent changes that could be anticipated preoperatively.

A linear regression model was constructed using both preoperative curvatures as predictors, yielding an accuracy of 0.75 with outliers and 0.93 after outlier removal for postoperative horizontal keratometry prediction. Similarly, there was an excellent prediction for postoperative vertical corneal curvature of 0.74. Still, after eliminating outliers, the accuracy increased from 0.06 with outliers to 0.74 without outliers, suggesting that outliers have a greater influence on vertical curvature. This suggested that vertical meridian responses might be more susceptible to patient-specific variability and biomechanical factors such as corneal rigidity and wound healing (Table 6).

**Table 6:** Performance metrics for postoperative K prediction using preoperative K values

N=42	MAE	MSE	RMSE	R-squared
Horizontal K prediction (outliers)	0.89	1.04	1.02	0.75
Vertical K prediction (outliers)	1.80	7.19	2.68	0.06
Horizontal K prediction (no outliers)	0.40	0.22	0.46	0.93
Vertical K prediction (no outliers)	0.89	0.99	0.99	0.74

Clinically, these results highlighted the stability of horizontal keratometry prediction, while vertical keratometry required a more cautious interpretation. From a methodological standpoint, the analysis illustrated the importance of addressing outliers in postoperative keratometry predictions. Extreme values had little influence on horizontal keratometry predictions but significantly distorted vertical keratometry predictions.

The main strength of this study was the use of a standardized scleral tunnel depth of 350 µm, which eliminated variability. In addition, the use of vector analysis with centroid calculation provided a more robust description of astigmatic changes than scalar representation alone. Limitations of the present study included a short follow-up period of one week. Long-term stability of the vector outcomes would strengthen conclusions about the sustained impact of scleral incision depth. In addition, although our regression analysis revealed important insights, the sample size was relatively modest, and larger cohorts would help confirm the generalizability of these findings.

## Conclusions

Temporal SICS with a standardized scleral tunnel depth of 350 µm produced a consistent early SIA, a modest centroid vector shift, and robust predictability of horizontal keratometry. The present results provided one of the first vector analyses of SIA outcomes reported with a precisely controlled tunnel depth.

Our regression analysis highlighted differences in the predictability of horizontal and vertical keratometric outcomes. Horizontal keratometry demonstrated a consistently strong association with its preoperative measurements, and outlier elimination did not materially alter the performance, underscoring the robustness of horizontal meridian prediction. In contrast, the vertical keratometry exhibited weaker predictive power at baseline but showed substantial improvement after outlier removal, suggesting greater sensitivity to extreme values.

This discrepancy likely reflected the architecture of temporal incision placement, with moderate depth primarily influencing the horizontal corneal meridian, leading to more uniform surgically induced changes and, hence, better predictability. Vertical meridian responses were comparatively more variable, possibly due to individual corneal biomechanics and differential wound-healing responses. Clinically, these findings emphasized that horizontal keratometric outcomes could be reliably anticipated, whereas vertical meridian prediction required careful consideration of outliers and patient-specific variability.

This work adds a unique dimension to the literature by explicitly linking a fixed tunnel depth to SIA vector and keratometric prediction. These results provided a platform for future research exploring scleral incision depth as a determinant of astigmatic outcomes. Future studies could evaluate the potential benefits of modifying tunnel depth, including whether deeper scleral incisions confer additional stability or whether more superficial incisions increase astigmatic variability. While our study did not compare multiple depths, the benchmark provided here serves as a reference for future prospective analyses aimed at optimizing scleral incision depth.

## References

[1] Mallik VK, Kumar S, Kamboj R, Jain C, Jain K, Kumar S (2012). Comparison of astigmatism following manual small incision cataract surgery: superior versus temporal approach. Nepal J Ophthalmol.

[2] Zawar SV, Gogate P (2011). Safety and efficacy of temporal manual small incision cataract surgery in India. Eur J Ophthalmol.

[3] Pattanayak S, Nanda AK, Swain AK (2022). Effect of depth of the sclerocorneal incision on postoperative corneal astigmatism in manual small-incision cataract surgery. Indian J Ophthalmol.

[4] Kumar N, Kaur G, Chadha C, Sethi N, Gupta NR, Gauri S (2022). Visual outcome after manual small-incision cataract surgery by viscoexpression technique. Indian J Ophthalmol.

[5] Pawar VS, Sindal DK (2012). A Comparative Study on the Superior, Supero-Temporal and the Temporal Incisions in Small Incision Cataract Surgeries for Post Operative Astigmatism. J Clin of Diagn Res.

[6] Rajappa SA, Bhatt H (2020). Minimizing surgically induced astigmatism in non-phaco manual small incision cataract surgery by U-shaped modification of scleral incision. Indian J Ophthalmol.

[7] Chatterjee T, Samaddar S, Bandyopadhyay SK (2017). Comparative study of comparison of surgically induced astigmatism (SIA) between the superior approach (incision) and temporal incision in small incision cataract surgery (SICS). J. Evolution Med. Dent. Sci.

[8] Patel K, Patel V, Patel N (2020). Comparison of surgical-induced astigmatism between sics superior and sics temporal. Indian J Clin Exp Ophthalmol.

[9] Rauf A, Ahmad F, Malik MB, Khan AM, Abbas H, Suri AK (2022). Comparison of Surgically Induced Astigmatism between Superior and Supero-Temporal Incisions in Manual Small Incision Cataract Surgery. Pak Armed Forces Med J [Internet].

[10] Reddy B, Raj A, Singh VP (2007). Site of incision and corneal astigmatism in conventional SICS versus phacoemulsification. Ann Ophthalmol (Skokie).

[11] Kavitha KNS, Kishore AK, Reddy GS, Jehan K (2017). Comparative study of surgically induced astigmatism in superior versus temporal incision in small incision cataract surgery cases. International Journal of Research in Medical Sciences.

[12] Jauhari N, Chopra D, Chaurasia RK, Agarwal A (2014). Comparison of surgically induced astigmatism in various incisions in manual small incision cataract surgery. Int J Ophthalmol.

[13] Gadikar AB (2020). A comparative study between superior, superotemporal and temporal incisions for corneal astigmatism after small incision cataract surgery with PCIOL. MedPulse International Journal of Ophthalmology.

[14] Goel R, Sontakke R, Shah S, Nagpal V, Kumar S, Koli O, Ojha S, Saini S, Arya D (2022). Correction of pre-existing astigmatism by on-axis incision size modulation in manual small-incision cataract surgery. Indian J Ophthalmol.

[15] Arthur E, Sadik AA, Kumah DB, Osae EA, Mireku FA, Asiedu FY, Ablordeppey RK (2016). Postoperative Corneal and Surgically Induced Astigmatism following Superior Approach Manual Small Incision Cataract Surgery in Patients with Preoperative Against-the-Rule Astigmatism. J Ophthalmol.

